# A statement about the publication describing genome sequencing of the Atacama skeleton

**DOI:** 10.1101/gr.237842.118

**Published:** 2018-05

*Genome Research* recently published a paper (Bhattacharya et al. 2018) describing the whole-genome sequencing of the Atacama skeleton. The manuscript underwent rigorous peer review by experts in evolutionary genetics and paleogenomics and the Editors of *Genome Research* stand behind the review process and the publication of this paper. The reported work has garnered significant public and scientific attention due to interest in the topic. At the same time, questions have been asked about the ethical standards that guided the investigation of the skeleton and the subsequent publication of the results (Nolan and Butte 2018).

*Genome Research* has an established track record of adherence to policies that protect human subjects in biomedical research. Current human subjects research policies do not typically cover the study of specimens of uncertain biological origins, such as the Atacama skeleton. The DNA sample from the Atacama skeleton did not qualify as human subjects research as defined by the Federal Office of Human Research Protections; thus, neither specific approval nor exemption was required for the study reported in the paper.

Nevertheless, the concerns about the study expressed by some Chilean scientists, their government, and some members of the public must be taken seriously. We also recognize the sensitivities related to the history and acquisition of the sample. The Editors and Publisher of *Genome Research* acknowledge that these issues require further discussion and agreement on rules of usage from within and outside the biological research community.

This experience highlights the evolving nature of this field of research, and has prompted our commitment to initiate community discussions aimed at establishing journal policies and author guidelines necessary for the publication of studies involving historical and ancient DNA samples.

The Editors and Publisher of *Genome Research*
